# Screening of more than 2000 Hungarian healthcare workers’ anti-measles antibody level: results and possible population-level consequences

**DOI:** 10.1017/S0950268818002571

**Published:** 2018-09-11

**Authors:** G. Lengyel, A. Marossy, N. Ánosi, S. L. Farkas, B. Kele, É. Nemes-Nikodém, V. Szentgyörgyi, I. Kopcsó, M. Mátyus

**Affiliations:** 1 Hungarian Defence Forces, Military Medical Centre, H-1134 Budapest, Robert Károly krt 44, Hungary; 2 University of Veterinary Medicine Budapest, H-1078 Budapest, István str. 2, Hungary; 3 Institute for Veterinary Medical Research, Centre for Agricultural Research, Hungarian Academy of Sciences, 1143 Budapest, Hungaria krt. 21, Hungary; 4 UK NEQAS for Microbiology, 61 Colindale Avenue, London, NW9 5EQ, UK

**Keywords:** Epidemics, healthcare workers, measles, nosocomial infection, seroepidemiology, vaccination

## Abstract

Due to the European measles epidemic and the increased number of imported cases, it can be theorised that the risk of exposure among Hungarian healthcare workers (HCWs) has increased. In 2017, the increased measles circulation in the region led to the emergence of smaller local and hospital epidemics. Therefore, our objective was to determine the herd immunity in the high-risk group of HCWs. A hospital-based study of detecting anti-measles IgG activity was performed in 2017 and included 2167 employees of the Military Medical Centre (Hungary). The screening of HCWs presented a good general seropositivity (90.6%). The highest seroprevalence value (99.1%) was found in the age group of 60 years or older. The lowest number of seropositive individuals was seen in the 41–45 years (86.2%) age group, indicating a significant herd immunity gap between groups. Regarding the Hungarian data, there might be gaps in the seroprevalence of the analysed HCWs, implying that susceptible HCWs may generate healthcare-associated infections. This study suggests that despite the extensive vaccination and high vaccine coverage, it is still important to monitor the level of protective antibodies in HCWs, or in a representative group of the whole population of Hungary, and possibly in other countries as well.

## Introduction

Despite the long-term and wide range measles eradication programme, there is still an ongoing epidemic in Europe. Along with the increased number of cases, the number of hospitalised patients with complications has risen as well [1], which has led to healthcare-associated transmission of measles, primarily driven by healthcare workers (HCWs) [1–4]. Since several susceptible HCWs have been infected despite of the proper protective measures (hand sanitation, surgical masks, protective gloves), introduction of vaccination among these employees has become the only effective way of preventing healthcare-associated spread [2–5].

Measles virus infection causes life-long protective immunity, and vaccination with two doses of measles, mumps and rubella vaccine (MMR) should give sufficient protection as well [6]. To reach and maintain herd immunity, the recommended vaccine coverage for the entire population needs to be approximately 95% or above [7]. Measles is an extremely contagious airborne virus with an estimated basic reproduction number (*R*_0_) of 12–18 [7]. This determines the herd immunity threshold, and therefore the vaccination coverage required to achieve elimination [8]. The critical immunisation threshold (*q*_c_) is between 95% based on the following formula: *q*_c_ = 1–1/*R*_0_ [7, 9].

Since 2016, the number of confirmed measles cases in Europe has been increasing, which is in correlation with the lower vaccine coverage [8, 10, 11]. In 2017, three minor measles outbreaks have been detected in Hungary, in which HCWs had been involved.

The first occurrences of measles were detected on 29 January 2017 and lasted until 10 March 2017 in Makó and Szeged. Among them, 54 cases were with measles-specific clinical symptoms [12]. Fifteen cases were confirmed (among them 13 HCWs) and the remaining 39 cases could be excluded according to laboratory analyses. Based on the sequencing of the viral RNA genome, five cases revealed genotype B3 (National Reference Laboratory for Measles and Rubella, National Public Health Institute, Budapest, Hungary), which were identical to the Romanian and Italian genotypes according to the data available of the MeaNS. The second group of imported cases were detected at the end of July 2017 in Nyíregyháza, Szabolcs-Szatmár-Bereg County, Hungary. Six unvaccinated Romanian children were admitted to hospital due to clinical signs of measles. These cases were also confirmed by the National Reference Laboratory for Measles and Rubella [13]. The disease could spread among the susceptible Hungarian population, as the measles virus infection of two HCWs was also confirmed [13].

Hungary participates in the measles eradication programme of the World Health Organization (WHO) since its development [8]. Vaccination against measles was introduced in Hungary in 1969 (containing formalin-inactivated virus particles) [14], and later, in 1984, a monovalent live measles vaccine was issued [14]. With the combined MMR vaccine introduced in 1991 [14, 15], the estimated vaccine coverage for the first-dose MMR vaccine in the period of 1997–2001 was close to 100% [15]. According to a recent report published by the European Centre for Disease Prevention and Control (ECDC), the vaccination coverage rate in several European countries, including Austria, Poland, UK, Czech Republic, Germany and Croatia, does not reach 95%, while in other countries, such as France, Italy and Romania, it is below 85% [16]. The majority of measles cases and the most severe outcomes were reported in the latter three countries [16]. Several European countries have reported the involvement of HCWs in the recent 2017 outbreak. In Italy, 315 cases among HCWs were reported, 67 in Greece, 35 in Belgium and 20 in the Czech Republic [16]. In Makó, Hungary there were 17 laboratory-confirmed measles cases, with 13 being diagnosed in HCWs [8, 17].

Compared with the average population, HCWs are exposed to a greater risk of infection [18], and they can play a significant role in transmitting the disease [19].

To resolve the contradiction between the high vaccination coverage and recently experienced minor outbreaks due to higher exposition of HCWs, this study aimed to measure the protection status of more than 2000 HCWs working in the frontline of the Hungarian healthcare system.

## Methods

### Ethical statement

The study proposal was reviewed and approved by the Institutional Ethical Board at Military Medical Centre, Budapest, Hungary in 2017. Written informed consent was obtained from the participants before blood sampling.

### Study population, measles history and estimated vaccine coverage

A screening in the Military Medical Centre, Budapest, Hungary was conducted in 2017. All HCWs of the institute, born before 1990 have been involved in the study. By the term ‘high-risk HCWs’ we mean doctors and nurses working in intensive care units, emergency departments and infectious diseases wards. Along with doctors and nurses, administrative and technical staff of these wards were screened as well.

During our study, a questionnaire survey was also conducted. Measles vaccination data were obtained by routine administrative assessment. More than 90% of participants did not know or was not sure about previous measles infection or vaccination history. Therefore, in the absence of proper documentation, these data were disregarded.

Our study included 2167 participants, employees of the Military Medical Centre (Hungary): among them 343 (15.8%) HCWs who worked at high-risk departments, 1186 (54.7%) at non-high-risk departments and 638 (29.4%) administrative and technical workers (mechanics, cafeteria, cleaning and laundry staff). Among them 368 medical doctors (16.9%) and 1161 nurses (53.5%) participated in this study. The mean age of HCWs was 47.1 years. Among the participants, 1736 were female (80.1%) and 431 were male (19.9%). Detailed parameters of participants are shown in Tables 1 and 2.

**Table 1.** Characteristics of the HCW population (*N* = 2167)

	Male *N* (%)	Female *N* (%)	Total *N* (%)
Total	431 (19.9)	1736 (80.1)	2167 (100)
Date of birth (age)			
After 1988 (below 30)	24 (1.1)	92 (4.2)	116 (5.4)
1988–1983 (30–35)	54 (2.5)	113 (5.2)	167 (7.7)
1982–1978 (36–40)	54 (2.5)	207 (9.6)	261 (12)
1977–1973 (41–45)	71 (3.3)	342 (15.8)	413 (19.1)
1972–1968 (46–50)	58 (2.7)	343 (15.8)	401 (18.5)
1967–1963 (51–55)	46 (2.1)	273 (12.6)	319 (14.7)
1962–1958 (56–60)	55 (2.5)	221 (10.2)	276 (12.7)
Before 1958 (60 or older)	69 (3.2)	145 (6.7)	214 (9.9)
Occupation			
Medical doctor	160 (7.4)	208 (9.6)	368 (16.9)
Nurse	123 (5.7)	1038 (47.9)	1161 (53.5)
Hospital unit			
High-risk departments	80 (3.7)	263 (12.1)	343 (15.8)
Non-high-risk departments	203 (9.4)	983 (45.4)	1186 (54.7)
Administrative and technical staff	148 (6.8)	490 (22.6)	638 (29.4)

**Table 2.** Measles seroprevalence in HCWs by distribution of departments and occupations

	Positive *N* (%)	Negative *N* (%)	Equivocal *N* (%)
Hospital departments			
High-risk departments	309 (90.08)^a^	26 (7.58)	8 (2.33)
Non-high-risk departments	1084 (91.39)^a^	67 (5.64)	35 (2.95)
Administrative and technical workers	571 (89.49)^a^	46 (7.21)	21 (3.29)
Occupation			
Doctor	342 (92.93)^b^	15 (4.07)	11 (2.99)
Nurse	1051 (90.52)^b^	78 (6.71)	32 (2.75)

Data presented as number (percentage) of HCWs in different groups.

a *χ*^2^ test, hospital departments, *χ*^2^ = 3.313, *P* = 0.5069.

b Δ*χ*^2^ test, occupation, *χ*^2^ = 3.439, *P* = 0.1791.

### Sample collection

A hospital-based study to detect anti-measles IgG activity was performed from February to May 2017. Collected blood samples were centrifuged and the sera were stored at −20 °C. The sera were tested after onefold thawing in all cases.

### Detection of measles-specific IgG antibodies

IgG antibody titre to measles virus was determined by a quantitative ELISA method (Serion ELISA classic Measles IgG, Institute Virion/Serion GmbH, Germany). The tests were performed using the EVOLIS Microplate System (Bio-Rad Laboratories, Inc., Hercules, CA, USA) according to the manufacturer's instruction. The Conformité Européene (European Conformity)/In Vitro Diagnostics (CE/IVD) validated kit permits detection of antibody activity in milli-international units per millilitres (mIU/ml), therefore comparison of results obtained in different laboratories was possible. The lower and upper limits of quantitation was declared as 50 and 5000 mIU/ml, respectively, from the manufacturer's kit insert. Results were declared Measles IgG-positive when the IgG level was above 200 mIU/ml, negative at <150 mIU/ml and equivocal between 150 and 200 mIU/ml. This diagnostic kit was validated using the second and third International Standard (IS) Sera of the WHO [20, 21].

### Statistics

The differences in seroepidemiological data among the item numbers of the groups were analysed with a *χ*^2^ test for independence. Calculating the *χ*^2^, a Yate's continuity correction was applied. Fisher's exact test was also performed. The calculations were done using GraphPadInStat V2 (GraphPad Software, V2.05a, USA). We applied the commonly used significance level, *P* < 0.05.

In the second part of our mathematical investigation, a factual amount of serum anti-measles IgG level was analysed. Kolmogorov–Smirnov and Shapiro–Wilk tests for normality were performed using STATISTICA Release 6.0 (StatSoft Inc., USA). In each age group, the probability distribution proved to be skew, not corresponding to the normal distribution. Because of failing normality criterion, a Kruskal–Wallis analysis of variance (ANOVA) on ranks with Dunn's post hoc multiple comparisons were used and performed with SigmaStat 3.0 (SPSS Inc., USA). The significance level of *P* < 0.05 has been applied, calculating with two-sided probabilities.

## Results and discussion

Previously, the immunity level of the institute's HCWs against morbilli virus was only estimated based on the average national vaccination coverage. Despite the fact that more than 99.5% of the population has been vaccinated receiving two doses of MMR within the confines of the national vaccination programme in Hungary, small outbreaks have occurred in the last year, primarily due to the importation of the virus from other countries in the last year [8]. Such outbreaks have not only affected unvaccinated people, but they also occurred in Hungarian HCWs in Makó, between those who had been previously vaccinated against measles [8]. Therefore, we aimed to investigate the level of anti-measles IgG antibodies in HCWs in our institute to estimate the herd immunity and the risk of infection in this group.

**Fig. 1.**
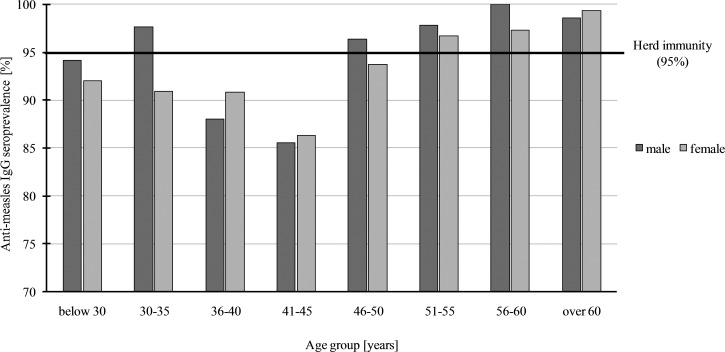
Measles seroprevalence in HCWs by gender and age groups in Military Medical Centre, Budapest, Hungary, 2017. There is a decrease in age group 36–45 in both genders which is considered significant, *χ*^2^ = 17 277; *P* = 0.0002 in men and *χ*^2^ = 45 748; *P* < 0.0001 in women. The horizontal line shows the herd immunity at 95%. In Hungary, the history of measles vaccination is the following: in 1969–1974 (44–49 years old) campaign vaccination in 9–23 months infants, with monovalent vaccine (Leningrad 16 strain); in 1974–1977 (41–44 years old) continuous vaccination of 10-month-old infants with monovalent vaccine (Leningrad 16 strain); 1978–1989 (29–40 years old) continuous vaccination of 14-month-old infants; 1989–1991 (27–29 years old) continuous vaccination of 14-month-old infants with monovalent vaccine (Rimevax) and revaccination at the age of 11 (Rimevax); 1991–1998 (20–29 years old) continuous vaccination of 15-month-old infants with trivalent vaccine (Pluserix) and revaccination at the age of 11 with monovalent vaccine (Rimevax).

The screening of 2167 HCWs presented a good general seropositivity in the population (90.6%). We sorted seroprevalence data according to eight age groups as follows: below 30, from 30 to 35, 36 to 40, 41 to 45, 46 to 50, 51 to 55, 56 to 60 and above 60 years, and the mean values obtained in these groups were 92.5%, 93.1%, 90.2%, 86.2%, 94.1%, 96.8%, 97.8% and 99.1%, respectively (seroprevalence data according to age and gender distribution are represented in Fig. 1).

**Fig. 2.**
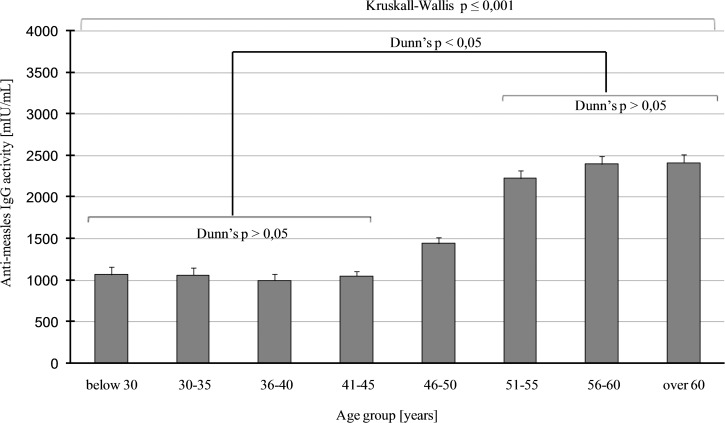
Measles total IgG levels, calculated in mIU/ml, using quantitative ELISA assay, are represented for each age group, with the mean value and the standard error. The upper long square braces represent the significant result of Kruskall–Wallis ANOVA with *H* = 441 693 and *P* ⩽ 0001. The origin of this significance is represented with the shorter square braces below, showing the simplified results of Dunn's post hoc multiple comparisons.

Seroprevalence data were different in the age groups, higher values above 95% (which is the critical immunisation threshold, *q*_c_) were observed in age groups of 51–55, 56–60 and above 60 years. Lower values were obtained in age groups of 41–45 (86.2%) and 36–40 years (90.2%). The lowest number of seropositive individuals, seen in the 36–40 and 41–45 years age groups, indicate a significant herd immunity gap in these two groups (Fig. 1). Consequentially, about 20% of HCWs between 41 and 45 years of age proved to be susceptible to the disease. This phenomenon has also been observed in other countries, with a similar vaccination strategy and results [22–29]. We could not find significant difference regarding seroprevalence between men and women. A Fisher's exact test result of *P* = 0.6963 was observed in people >40 years old and was non-significant. Regarding people between 40 and 45 and above 40 years, the same test results *P* = 0.8482 and *P* = 0.6963 in this order (both non-significant) were observed. Comparison of data of employees with higher and lower qualifications and different departments (high-risk, non-high-risk, administrative and technical staff) revealed that there was no significant difference (*χ*^2^ = 2.594; *P* = 0.2733). By using a different statistical method for the same comparison, a non-significant result was found (Fisher's exact test *P* = 0.0863). Regarding the average anti-measles IgG levels of HCWs older than 50 years, they had more than twice as much antibodies as people younger than 46 (Kruskall–Wallis *H* = 441.693; *P* ⩽ 0.001; Fig. 2); this age group probably represents the naturally infected people.

Due to the progressing European measles epidemic, the number of imported cases has increased, and raised the risk of exposure to HCWs [8, 10, 11]. The risk is predominantly in the emergency department, infectious diseases ward and intensive care unit, where HCWs are in close contact with potentially infectious patients. In most cases, patients with measles infection visit hospitals before the onset of rash, when the virus is highly contagious [8] and in the absence of characteristic symptoms, measles infection is rarely suspected at this point [30]. Based on our data, no significant difference could be observed in seroprevalence, regarding people working at high-risk departments or other areas, most probably due to mandatory vaccination in Hungary.

Infection of susceptible HCWs can induce healthcare-associated transmission of measles cases, and this way they can further spread the infection in hospitals [8, 30]. HCWs can serve as reservoirs of several pathogens [31]. Additional factors that might contribute to healthcare-associated infections are vaccination of HCWs, the workers’ and patients’ access to disinfection, and HCWs staying at work when ill [32]. According to a study, the HCWs’ adherence to hygiene guidelines is not appropriate [33]. Measles virus is capable of staying infective for a long period of time in aerosol suspension, making the transmission more facile [34, 35]. Moreover, healthcare-associated measles infections are more severe, and lead to complications more often in already immunocompromised patients [36]. Because of the considerable scale of contagiosity, the main driving force of measles outbreaks depends on the number of susceptible workers [7]. Therefore, the main solution to prevent major outbreaks is to keep the number of unprotected persons as low as possible, by keeping track of the vaccination and previous measles infection of HCWs. If decent documentation is not available, HCWs should take a booster MMR vaccination.

In Hungary, measles epidemics occurred during 1973–1974, 1980–1981, 1988–1989 and 2016–2017 years, respectively [37]. Since vaccination started in 1969, gaps in the immunity of the population can be explained by primary and secondary vaccine failure, which can occur due to vaccine- or host-related causes. Development of the adequate immune response may be hampered by several factors, such as the presence of maternal antibodies at the first vaccination in children, immunosenescence, low nutrition, obesity, allergy etc. [38].

The quality of the vaccine might have been deteriorated during production, transport and handling in the early years of history of measles vaccination. Vaccination with only one dose might be insufficient because of the above-mentioned factors; therefore, immunisation with two doses of MMR was introduced, in order to avoid epidemics. Monitoring measles IgG antibody titre of HCWs is necessary to evaluate the possible need for booster vaccination, because the vaccine-induced measles IgG antibody titre is naturally decreasing with an approximately 5.6% per year rate even after the second dose of MMR [39].

Our results indicate a high level of total measles IgG activity as a result of the vaccination against measles. Since the strict and mandatory vaccination programme, Hungarian population has more than 99% vaccine coverage after the first dose. Sporadic small outbreaks over the last years have occurred solely due to imported virus [8, 14].

The vaccination of these HCWs occurred decades ago; therefore, we do not know the exact circumstances of the immunisation of these workers. The gap in herd immunity might be caused by improper handling of the vaccine application of smaller doses due to the high reactivity of the vaccine, or the immunisation of infants younger than 10–12 months (maternal antibodies could interfere with the immunisation process) [40].

## Conclusion

This study demonstrates that despite the extensive vaccination and proposed high vaccine coverage, it is important to monitor the level of protective antibodies in a larger part of HCWs or in a representative group of the whole population in Hungary, and possibly in other countries as well. Regarding the Hungarian data, there might be gaps in the seroprevalence of the examined HCWs. The gaps in herd immunity of HCWs may lead to healthcare-associated epidemics; therefore, in the absence of well-documented vaccination data, we recommend all HCWs to be (re-)vaccinated with MMR to ensure their proper protection. Extremely high titres were found in people who were born before the vaccination era (before 1969), suggesting natural infection-induced life-long immunity. The mainstay of our conclusions was based on the relatively high number of screened HCWs compared with similar studies.

In a large, regional medical centre, like ours, all proper protective measures should be taken to avoid any disturbance in the continuous patient care caused by an easily preventable disease.
